# Memoirs of the Royal Academy of Sciences of Sweden for the Year 1777

**Published:** 1782

**Authors:** 


II. *Kongl. Vetenskaps Academiens Handlingar för ar* 1777. i e. *Memoirs of the Royal Academy of Sciences of Sweden for the Year* 1777. 8vo, Stockholm.

1. *Observations to shew that the loss of one or other of the external senses is compensated by a greater perfection of the rest. By* R. Martin, M.D. *Pro-*

*Profeſſor of Anatomy at Stockholm.*—In the courſe of this paper we meet with ſeveral curious facts. The moſt remarkable of theſe is our author's account of a Swediſh peaſant thirty-four years old, who though blind from his infancy, has acquired conſiderable dexterity in ſeveral of the mechanic arts. He not only eaſily finds his way alone through the foreſts to cut wood, but makes a variety of utenſils in iron, wood, &c. likewiſe ſhoes, needle-work, &c. He plays at cards, but he cannot diſtinguiſh colours. Another blind man, who is a native of Finland, is ſpoken of as excelling in all kinds of fine wood work. Our author makes mention of a woman whom he couched, and who for ſome time after the operation, whenever the bandage was removed, could not ſee, unleſs the window-curtains of the room were drawn. In a ſecond paper on the ſame ſubject, Profeſſor Martin ſpeaks of a man who being deprived of the uſe of his hands, performs all the uſual functions of the hands with his feet; and of another perſon now living at Stockholm, who having likewiſe loſt the uſe of his hands, has accuſtomed himſelf to write with his mouth.

II. *An account of the ſtate of population in four diſtricts of Swediſh Lapland. By* Mr. Hollſten.— Each of theſe diſtricts is ſaid to contain upwards of 100 ſquare miles, and yet the total number of inhabitants does not exceed 3370. They are ſometimes viſited by the ſmall-pox, their dread of which is ſo great, that when a perſon is attacked with it, they generally leave him to himſelf. In another paper on the ſame ſubject, Mr. Hogſtroins endeavours to prove that Lapland was formerly more populous than it is at preſent.

III. *Obſervations on the ſalt-petre manufactory at Helſingfors. By* Mr. Berger.—This paper contains ſeveral remarks on the production, conſtituent parts, and method of refining ſalt-petre. Our author obſerves that the acid of nitre is intimately combined with a diluted oily ſubſtance, which prevents it from ſhooting into chryſtals. It is not the practice, it ſeems, in Sweden to procure ſalt-petre by the addition of an alkali; but the common ſalt and oily matter, as well as the calcareous earth united with the latter, are ſeparated by repeated ſolutions and evaporations. We are told that at Helſingfors there is a ſpring which contains nitre in the proportion of half an ounce to eight pounds of the water.

IV. *An*

IV. *An account of a fatal case of hydrophobia. By* J. L. Odhelius, M.D.—We have here an instance of a disease which is said to be very uncommon in Sweden. The patient was a boy 12 years old, who had kissed a sick dog that afterwards proved to be mad. The patient had taken the animal to-bed with him the night before it died. There was no appearance of any wound, and he denied that he had been bit. When the symptoms of hydrophobia came on, recourse was had to venæsection, but the blood that was taken away exhibited no inflammatory crust. Mercurial frictions, opium, and musk were given freely, but without effect, as the patient died. The dead body soon became putrid.

V. *The case of a young woman who was bit by a dog. By* Martin Stutzer.—This paper relates to the treatment of a maid servant who was bit in two places by a dog that was supposed to be mad. Cantharides in powder were applied, so as to produce a discharge from the wounds; salivation was excited by mercurial frictions, and the patient remained free from infection. Nothing, however, can be more suspicious than the prophylactics recommended against the hydrophobia, even in cases where the madness of the

 animal animal is afcertained beyond a doubt. In the present inftance, it does not clearly appear that the animal itfelf was mad, and of courfe the cafe can be of no ufe.

VI. *An account of the inefficacy of foap and lime water in a cafe of calculus. By* P. J. Bergius, M.D. *Profeffor of Natural Hiftory and Chemiftry at Stockholm.*—The patient, whofe cafe is here related, took every day, during fourteen years, half an ounce of foap, and three pints of lime water. After death a calculus was found in the bladder that weighed eleven ounces. The author obferves that he has tried the fame remedy in cafes of gall ftone, but without any good effect. In three inftances of the latter difeafe, the patient found relief from the ufe of cream of tartar.

Of the other papers contained in this volume, we fhall content ourfelves with enumerating the general heads, viz. 1. Experiments on fea water, by Sir Thorb. Bergman. (See Vol. I. of our Journal, page 74) 2. Obfervations to prove that the magnefia of nitre is not a calcareous earth; and 3. Remarks on Platina, and the *lapis hydropha-phanus*, or *oculus mundi*, by the fame: all thefe have been fince reprinted in his *Opufcula Chemica.* 4. An account of the worms that are found found in wheat, and the means of deſtroying them, by Mr. Bierkander; 5. A deſcription, with engravings, of the *Protea Sceptrum Guſtavianum*, and 6. *Limex Paradoxus*, two plants, and of a new animal diſcovered at the Cape by Dr. Sparmann; 7. Experiments with the Electrophorus; and 8. Obſervations on the declination of the needle at Stockholm; 9. An account of a wound of the femoral artery ſucceſsfully treated by Olaus Acrel, M. D. &c. (ſee our preſent volume, page 20) with an appendix by Profeſſor Martin, pointing out the caſes of aneuriſm which may be cured by preſſure, and thoſe in which a ligature is neceſſary; 10. A deſcription of the *Hydnora Africana*, accompanied with an engraving of the fruit, by Dr. Thunberg; 11. Remarks on the draining of marſhes, by Mr. Gadd; 12. A deſcription of the *Gentiana Saxoſa*, by Geo. Forſter. 13. Remarks on the management of bees, by Mr. Ahlgren; 14. on the culture of potatoes, by J. Alſtroemer; 15. An account of an African animal of the fox kind, by Mr. Skoldebrand; 16. Obſervations on the declination of the needle in ſeveral places within the polar circle, by Mr. Hellant; 17. A deſcription and engraving of the *Pinus Viminalis, ramulis elongatis,*

*gatis*, *dependentibus*, *flexuosis*, *laxis*, a new species of pine that grows in Upland and Südermamland, by Baron Clas Alströmer, and Dr. Sparrmann. 18. Observations on the *Lapis hydrophanus*, or *oculus mundi*, in several papers, by Messrs Pötzsch, Qwist, Veltheim, A. Murray, and Brümich.

